# C17orf75 (Njmu-R1) promotes hepatocellular carcinoma progression: a pan-cancer analysis and experimental validation

**DOI:** 10.3389/fimmu.2026.1828437

**Published:** 2026-07-09

**Authors:** Hao Liang, Nanbin Liu, Yibing Melody Zhai, Shiyan Guo, Ke Du, Kun Li, Xiaolin Zhu

**Affiliations:** 1 Department of Laboratory Medicine, The Second Xiangya Hospital, Central South University, Changsha, Hunan, China; 2 Department of General Surgery, The Second Affiliated Hospital of Xi’an Jiaotong University, Xi’an, China; 3 Department of Ultrasound Medicine, The Third Xiangya Hospital of Central South University, Changsha, Hunan, China

**Keywords:** C17orf75, hepatocellular carcinoma, pan-cancer analysis, prognosis, tumor microenvironment

## Abstract

**Background:**

Chromosome 17 Open Reading Frame 75 (C17orf75) encodes the protein Njmu-R1 (Protein Njmu-R1),, which is involved in intracellular vesicle trafficking; however, its role in tumor progression remains largely unclear.

**Methods:**

Public datasets from The Cancer Genome Atlas (TCGA), Gene Expression Omnibus (GEO), and the Human Protein Atlas (HPA) were analyzed to evaluate the expression profile, mutation landscape, and diagnostic and prognostic value of C17orf75. Bioinformatics analyses were subsequently performed to explore its associations with immune infiltration. In addition, functional assays were conducted on Hep3B and MHCC-97H cells, and immunohistochemistry (IHC) was performed on clinical liver hepatocellular carcinoma (LIHC) samples.

**Results:**

C17orf75 was significantly upregulated in multiple cancer types, particularly in LIHC. Elevated C17orf75 expression was associated with unfavorable prognosis and advanced clinicopathological features in LIHC. Functional enrichment analyses indicated that C17orf75-related genes were involved in cell cycle regulation, DNA replication, and epithelial–mesenchymal transition (EMT). Furthermore, C17orf75 expression was closely correlated with immune infiltration, ferroptosis-related genes, and m6A regulators. Knockdown of C17orf75 inhibited the proliferation, migration, and invasion of LIHC cells. C17orf75 knockdown induced G2-phase arrest without significantly affecting apoptosis. Moreover, knockdown of C17orf75 suppressed EMT.

**Conclusion:**

C17orf75 plays an important role in LIHC progression by regulating cell cycle progression and EMT, and it may serve as a potential therapeutic target for LIHC.

## Introduction

It is estimated that by 2026, approximately 2,114,850 new cancer cases will occur in the USA (1). Similarly, cancer imposes a substantial health and socioeconomic burden in China. The incidence and mortality rates of lung, breast, colorectal, and prostate cancers have increased rapidly, whereas the prevalence of liver, stomach, esophageal, and cervical cancers remains relatively high (2).

In China, more than 360,000 new liver hepatocellular carcinoma (LIHC) cases are diagnosed each year, resulting in approximately 320,000 deaths annually (3). Currently, histopathological examination remains the standard for the diagnosis of LIHC. However, this approach has several limitations, including dependence on subjective interpretation, time-consuming procedures, and potential interobserver variability (4). Moreover, LIHC is associated with poor prognosis, with only 5%–15% of patients diagnosed at an early stage being eligible for surgical resection. For patients with advanced disease, treatment options include transarterial chemoembolization (TACE) and systemic therapies such as sorafenib. Nevertheless, these treatments are often accompanied by considerable adverse effects and limited therapeutic efficacy (5). Therefore, identifying new diagnostic biomarkers and therapeutic targets for LIHC remains an urgent priority.

Chromosome 17 Open Reading Frame 75 (C17orf75) is a protein-coding gene that encodes the protein Njmu-R1 (also known as SRI2), which has been implicated in several diseases, including congenital achromatopsia type 1 (6, 7). Njmu-R1 interacts with TBC1D23 (TBC1 domain family member 23) and participates in the capture of AP-1-derived vesicles mediated by the Golgi apparatus, potentially contributing to intracellular vesicle trafficking and spermatogenesis (8). The Njmu-R1 protein encoded by C17orf75 is mainly localized in cytoplasmic vesicles and the trans-Golgi network, suggesting a role in intracellular protein transport and vesicle–Golgi interactions (9). Homologs of C17orf75 are widely conserved across multiple species, including mice, chickens, lizards, zebrafish, and *Xenopus laevis* (10, 11). Despite these findings, the role of C17orf75 (Njmu-R1) in human cancers has not yet been comprehensively investigated.

Despite increasing interest in C17orf75 and its encoded protein Njmu-R1, its functional role and biological mechanisms in LIHC progression remain unclear. In particular, whether C17orf75 contributes to coordinated regulation of proliferation and metastasis-related processes has not been investigated. In this study, we investigate the expression patterns, diagnostic value, and prognostic significance of C17orf75 using datasets from The Cancer Genome Atlas (TCGA), Gene Expression Omnibus (GEO), and Human Protein Atlas (HPA). Furthermore, we explored the associations between C17orf75 and clinicopathological characteristics, gene mutations, protein–protein interaction (PPI) networks, differentially expressed genes (DEGs), and immune infiltration. Molecular docking and drug sensitivity analyses were performed to evaluate potential therapeutic implications. Finally, functional experiments were conducted to validate the biological role of C17orf75 in LIHC cells.

## Methods

### Data acquisition and preprocessing

Gene expression profiles together with associated clinical annotations were retrieved from publicly available cancer resources, primarily TCGA and GEO. When normal control samples were insufficient in TCGA, additional datasets were incorporated to ensure adequate comparison. Information on protein expression and subcellular distribution was obtained from curated protein databases. To further delineate the expression characteristics, spatial transcriptomics and single-cell RNA sequencing datasets were also integrated. Prior to analysis, all data underwent standard preprocessing and normalization procedures.

### Bioinformatics analysis

Computational analyses were conducted using R to evaluate gene expression differences, clinical relevance, and survival outcomes. Functional interpretation of genes associated with C17orf75 was performed using enrichment strategies. The tumor immune microenvironment, along with immune-related signatures, was characterized using established analytical frameworks and publicly available immunological datasets. Furthermore, genomic alterations, coexpression relationships, and protein interaction networks were examined to investigate potential biological functions. Drug response and immunotherapy efficacy were estimated using pharmacogenomic modeling approaches, and candidate compounds were subsequently assessed through molecular docking.

### Clinical samples

Twenty pairs of LIHC specimens, including paired tumor and adjacent nontumorous tissues as well as formalin-fixed paraffin-embedded samples, were collected for validation experiments.

### Cell culture and transfection

Hep3B (Human hepatic carcinoma 3B cells) and MHCC-97H (Human high metastatic liver cancer cells 97) cell lines and normal liver cells were maintained under conventional culture conditions. Transient knockdown of C17orf75 was achieved via small interfering RNA (siRNA) transfection, with nontargeting sequences used as controls.

### Quantitative real-time PCR and Western blotting

mRNA expression levels were quantified using real-time PCR, whereas protein abundance was assessed by Western blot analysis.

### Functional assays

Cellular proliferative capacity, as well as migratory and invasive behaviors, were examined using standard *in vitro* assays. Flow cytometry was additionally employed to evaluate cell cycle progression and apoptotic activity.

### Statistical analysis

Data analyses were performed using R and GraphPad Prism software. Statistical comparisons were selected according to data distribution and experimental design. Survival analyses were conducted using Kaplan–Meier estimation and Cox proportional hazards models. A two-tailed *p*-value below 0.05 was considered indicative of statistical significance.

### Supplementary information

Extended methodological details, including experimental procedures, analytical parameters, reagent specifications, and computational pipelines, are provided in the **Supplementary Methods** section (12–35).

## Results

### Expression of C17orf75 in normal human tissues

C17orf75 mRNA is widely expressed across human tissues (**Supplementary Figure 1A**). C17orf75 is primarily expressed in the parathyroid gland, testis, retina, kidney, choroid plexus, heart muscle, epididymis, skeletal muscle, cerebellum, liver, and thymus (**Supplementary Figures 1B–D**).

The protein encoded by C17orf75 (Njmu-R1) is broadly expressed across tissues, with relatively high levels in the cerebellum, thyroid gland, bronchus, liver, pancreas, kidney, ovary, and breast (**Supplementary Figure 1E**).

As shown in **Supplementary Figure 1F**, Njmu-R1 protein expression exhibits notable heterogeneity across different tissues, with particularly high levels in testicular germ cells and cardiac myocytes, as well as in epithelial cells of organs such as the liver and kidney.

Immunocyte analysis revealed that C17orf75 is enriched in basophils, naïve B cells, and NK cells, indicating relatively low immune cell specificity (**Supplementary Figure 1G**).

We further analyzed subcellular localization and protein structural features of Njmu-R1 (**Supplementary Figures 1H, I**). Njmu-R1 is localized in the plasma membrane and cytosol (**Supplementary Figure 1J**).

### C17orf75 is upregulated in multiple cancer types

**Figure 1:**
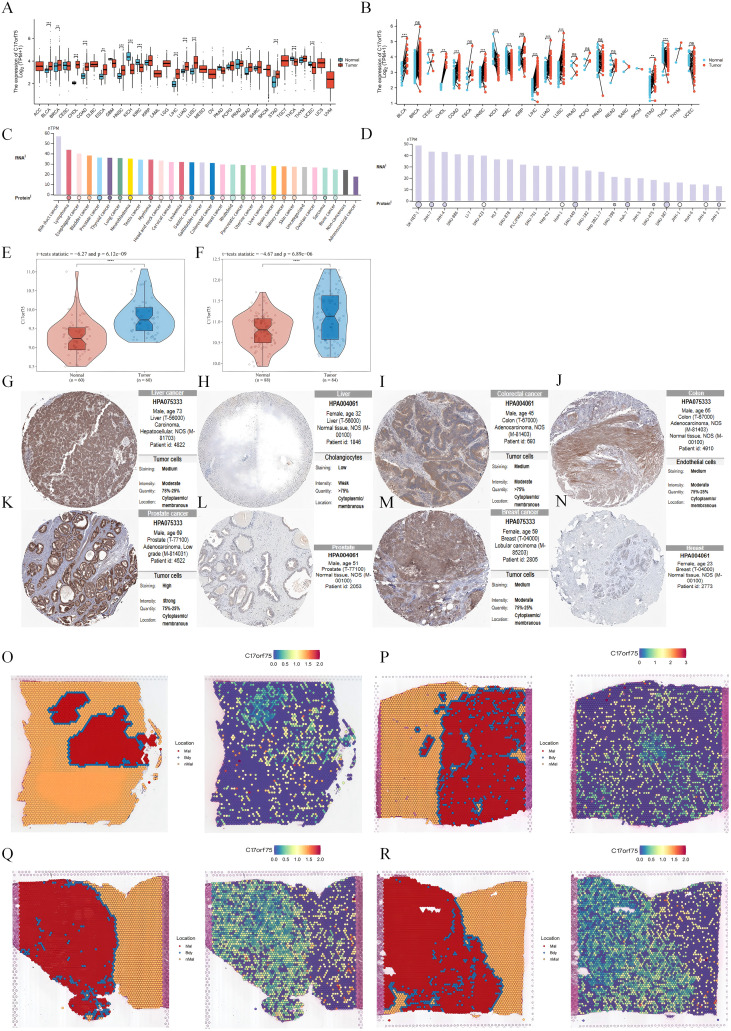
C17orf75 expression profiling. **(A, B)** C17orf75 expression in unpaired and paired pan-cancer tissues from the TCGA database. **(C)** mRNA expression of C17orf75 in various pan-cancer cell lines. **(D)** mRNA expression of C17orf75 in LIHC cell lines. **(E, F)** C17orf75 mRNA expression in LIHC based on the GEO datasets GSE19804 and GSE37182. **(G–N)** HPA database IHC staining showing Njmu-R1 protein expression in LIHC, colorectal carcinoma, prostate carcinoma, and breast carcinoma. **(O–R)** C17orf75 expression in HCC_JH-HCC_P8, HCC_Pmid35673582-HCC1, HCC_Pmid35673582-HCC2, and HCC_Pmid35673582-HCC3 tissue. (^*^p < 0.05; ^**^p < 0.01; ^***^p < 0.001).

C17orf75 was elevated across 15 unpaired and 11 paired malignant tissues (**Figures 1A, B**). Moreover, C17orf75 mRNA was principally expressed in bile duct cancer and esophageal cancer cell lines, while the Njmu-R1 protein was abundant in rhabdoid, prostate cancer, and neuroblastoma cell lines (**Figure 1C**).

Focusing on LIHC, C17orf75 was enriched in SK-HEP-1, SNU-449, and SNU-387 cell lines (**Figure 1D**). Consistently, analysis of GEO datasets confirmed that C17orf75 is significantly upregulated in lung adenocarcinoma (LUAD) and colorectal adenocarcinoma (COAD) (**Figures 1E, F**).

To further investigate protein expression patterns, IHC staining images were analyzed, revealing that Njmu-R1 protein levels are higher in liver, colon, prostate, and breast cancer tissues (**Figures 1G–N**).

Spatial transcriptomics analysis demonstrated that C17orf75 is predominantly expressed in tumor regions in LIHC (**Figures 1O–R**).

### Genetic alterations of C17orf75 in pan-cancer

To evaluate genetic alterations of C17orf75 across pan-cancer, we performed a comprehensive analysis using cBioPortal and found that C17orf75 alterations occurred in approximately 5% of cases (**Supplementary Figure 2A**).

Further analysis revealed that the highest alteration frequencies of C17orf75 were observed in bladder cancer, embryonal tumors, and endometrial cancer (**Supplementary Figure 2B**). Notably, amplification was identified as the most common type, while a substantial proportion of alterations in endometrial cancer were deletions.

Mutation analysis identified six specific alteration sites (**Supplementary Figure 2C**). Copy number variation (CNV) analysis showed that C17orf75 was predominantly characterized by heterozygous amplification (**Supplementary Figure 2D**).

We further characterized the CNV landscape of C17orf75, including both homozygous (**Supplementary Figure 2E**) and heterozygous (**Supplementary Figure 2F**) amplification and deletion events. In addition, single-nucleotide variant (SNV) analysis demonstrated that the highest mutation frequencies were present in UCEC, SKCM, and CESC (**Supplementary Figure 2G**). A total of 63 SNVs were identified, including 58 missense mutations and 5 deletions, which were further classified into transitions (Ti) and transversions (Tv) (**Supplementary Figures 2H, I**).

In LIHC, C17orf75 mutations were relatively rare, with four mutation sites identified, all of which were missense mutations (**Supplementary Figure 2J**). Oncoplot analysis indicated that highly mutated genes such as TP53, TTN, and CTNNB1 were predominantly characterized by missense mutations, and mutation frequencies were higher in the C17orf75 high-expression group (**Supplementary Figure 2K**).

We further summarized the top 10 genes ranked by mutation frequency, along with their mutation types and SNV classifications (**Supplementary Figure 2L**).

### Diagnostic value of C17orf75 in pan-cancer

C17orf75 exhibited strong diagnostic performance across multiple cancer types, including COAD (area under the curve [AUC] = 0.869), LIHC (AUC = 0.917), lung squamous cell carcinoma (LUSC) (AUC = 0.842), and stomach adenocarcinoma (STAD) (AUC = 0.825) (**Supplementary Figure 3A**).

Furthermore, C17orf75 had good predictive performance for 1-, 3-, and 5-year overall survival (OS) in these cancers (**Supplementary Figure 3B**).

### Prognostic value of C17orf75 in pan-cancer

**Figure 2:**
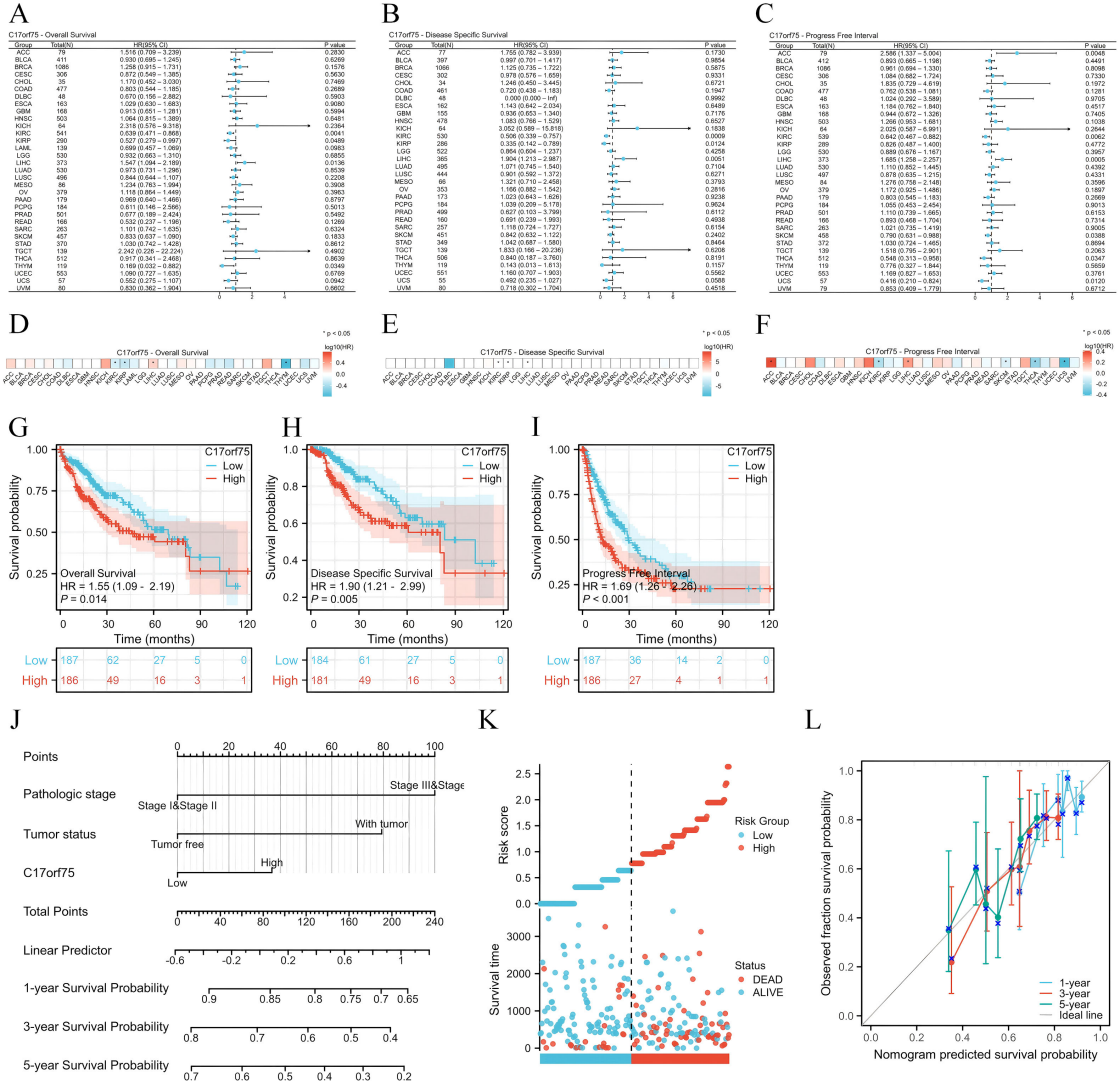
Prognostic value of C17orf75 in pan-cancer. **(A–F)** Forest plots and prognostic heatmaps of C17orf75 in pan-cancer. **(G–I)** KM curves of C17orf75 in LIHC. **(J)** Prognostic nomogram incorporating C17orf75 in LIHC. **(K)** Distribution of survival status according to C17orf75 expression levels. **(L)** Prognostic calibration curves corresponding to C17orf75.

Multivariate Cox regression analyses were performed to evaluate C17orf75’s association with OS, DSS, and PFI. Forest plots (**Figures 2A–C**) and heatmaps (**Figures 2D–F**) demonstrated the prognostic relevance of C17orf75 across pan-cancer. High C17orf75 expression was significantly associated with poor prognosis in LIHC (**Figures 2G–I**).

Multivariate Cox regression analysis identified tumor status (*p* = 0.009) and high C17orf75 expression (*p* = 0.034) as independent prognostic factors for OS (**Table 1**). A prognostic nomogram was constructed (**Figure 2J**). The risk score showed that mortality increased with higher risk scores, and patients with elevated C17orf75 expression had a higher risk of death (**Figure 2K**). Calibration plots indicated strong concordance between estimated and actual survival probabilities at 1-, 3-, and 5-year time points (**Figure 2L**).

**Table 1:** Univariate and multivariate Cox regression analysis of clinicopathological characteristics associated with LIHC overall survival.

Characteristics	Total (*N*)	Univariate analysis		Multivariate analysis	
		Hazard ratio (95% CI)	*p*-value	Hazard ratio (95% CI)	*p*-value
Pathologic T stage	370				
T1	183	Reference		Reference	
T2	94	1.431 (0.902–2.268)	0.128	1.519 (0.828–2.784)	0.177
T3	80	2.674 (1.761–4.060)	**< 0.001**	2.374 (0.304–18.515)	0.409
T4	13	5.386 (2.690–10.784)	**< 0.001**	4.480 (0.493–40.687)	0.183
Pathologic N stage	258				
N0	254	Reference			
N1	4	2.029 (0.497–8.281)	0.324		
Pathologic M stage	272				
M0	268	Reference		Reference	
M1	4	4.077 (1.281–12.973)	**0.017**	0.856 (0.163–4.500)	0.854
Pathologic stage	349				
Stages I and II	259	Reference		Reference	
Stages III and IV	90	2.504 (1.727–3.631)	**< 0.001**	1.126 (0.149–8.521)	0.909
Residual tumor	344				
R0	326	Reference			
R1 and R2	18	1.604 (0.812–3.169)	0.174		
Histologic grade	368				
G1	55	Reference			
G2	178	1.162 (0.686–1.969)	0.576		
G3	123	1.185 (0.683–2.057)	0.545		
G4	12	1.681 (0.621–4.549)	0.307		
Gender	373				
Women	121	Reference			
Men	252	0.793 (0.557–1.130)	0.200		
Age	373				
≤ 60	177	Reference			
> 60	196	1.205 (0.850–1.708)	0.295		
Race	361				
Asian	159	Reference			
Black or African American	17	1.585 (0.675–3.725)	0.290		
White	185	1.323 (0.909–1.928)	0.144		
Tumor status	354				
Tumor free	202	Reference		Reference	
With tumor	152	2.317 (1.590–3.376)	**< 0.001**	1.870 (1.170–2.989)	**0.009**
BMI	336				
≤ 25	177	Reference			
> 25	159	0.798 (0.550–1.158)	0.235		
AFP (ng/ml)	279				
≤ 400	215	Reference			
> 400	64	1.075 (0.658–1.759)	0.772		
Prothrombin time	296				
≤ 4	207	Reference			
> 4	89	1.335 (0.881–2.023)	0.174		
Albumin (g/dl)	299				
< 3.5	69	Reference			
≥ 3.5	230	0.897 (0.549–1.464)	0.662		
Child–Pugh grade	240				
A	218	Reference			
B and C	22	1.643 (0.811–3.330)	0.168		
Fibrosis Ishak score	214				
0	75	Reference			
1/2, 3/4, 5, and 6	139	0.772 (0.465–1.281)	0.316		
Vascular invasion	317				
No	208	Reference			
Yes	109	1.344 (0.887–2.035)	0.163		
Adjacent hepatic tissue inflammation	236				
None	118	Reference			
Mild	101	1.204 (0.723–2.007)	0.476		
Severe	17	1.144 (0.447–2.930)	0.779		
C17orf75	373aa	1.471 (1.103–1.963)	**0.009**	1.489 (1.031–2.149)	**0.034**

Bold values indicate statistical significance (P < 0.05).

### Association between C17orf75 expression and clinicopathological features in LIHC

**Figure 3:**
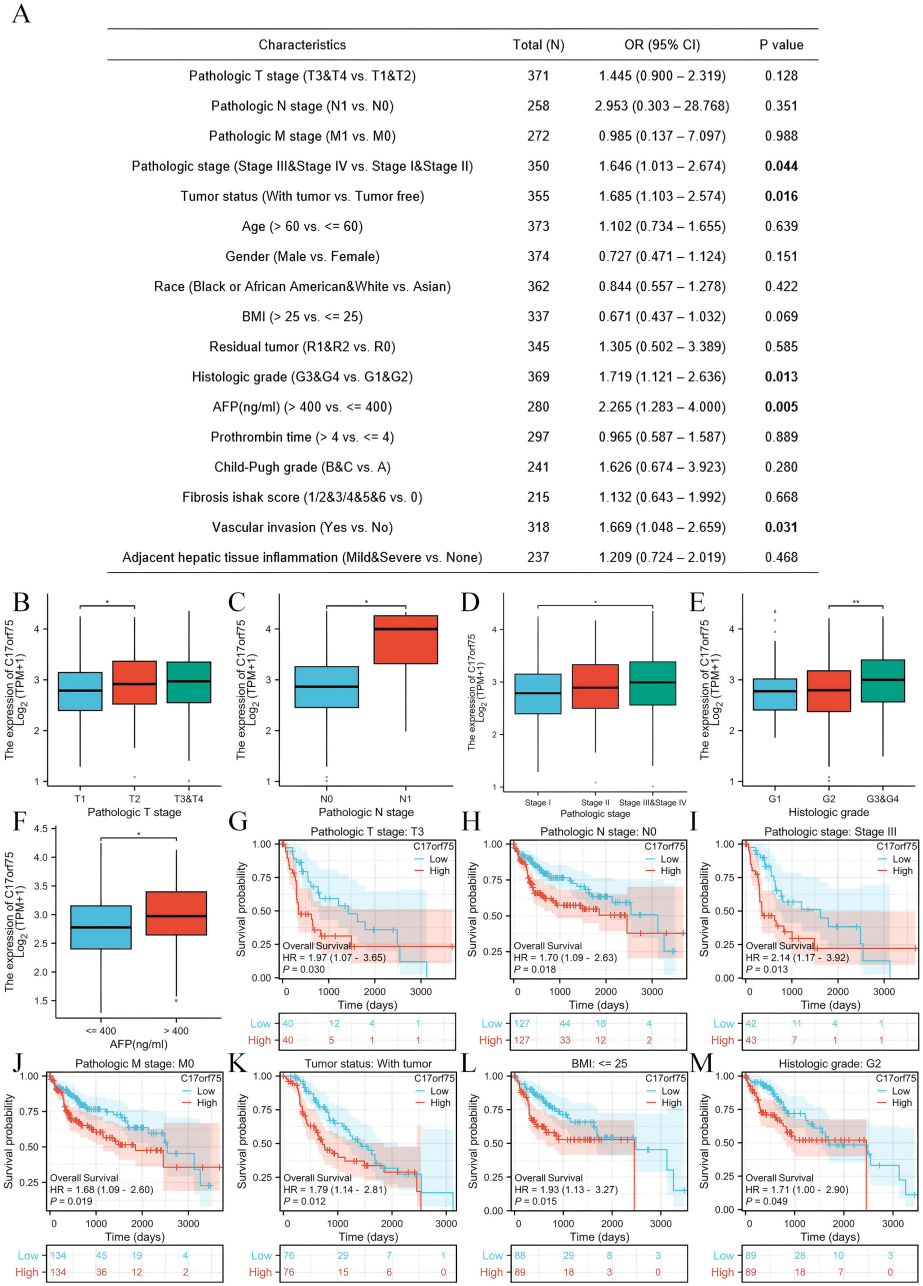
Prognostic value and clinical relevance of C17orf75 in LIHC subgroups. **(A)** Logistic analysis of C17orf75 in LIHC. **(B–F)** Relationship between C17orf75 expression and pathological T stage, pathologic N stage, pathologic stage, histologic stage, and AFP in LIHC. **(G–M)** Prognostic significance of C17orf75 in LIHC subgroups. (^*^p < 0.05; ^**^p < 0.01).

High C17orf75 expression was significantly associated with advanced pathologic stage (odds ratio [OR] = 1.646, *p* = 0.044), tumor presence (OR = 1.685, *p* = 0.016), higher histologic grade (OR = 1.719, *p* = 0.013), and AFP levels > 400 ng/mL (OR = 2.265, *p* = 0.005) (**Figure 3A**).

Elevated C17orf75 expression was correlated with advanced pathologic T stage, N stage, overall stage, histologic grade, and AFP level in LIHC (**Figures 3B–F**).

Subgroup analysis demonstrated that high C17orf75 expression remained significantly associated with poor prognosis across multiple subgroups (**Figures 3G–M**).

### Association of C17orf75 with ferroptosis and m6A methylation in LIHC

C17orf75 showed positive correlations with most m6A-related genes, including METTL3 (Methyl transferase like protein 3), METTL14, WTAP, VIRMA, and RBM15, whereas no significant correlation was observed with ALKBH3 (**Supplementary Figure 4A**).

C17orf75 was positively correlated with multiple ferroptosis-related genes, including CDKN1A, CISD1, EMC2, etc. Notably, it showed a negative correlation with MT1G (**Supplementary Figure 4B**), suggesting a potential role in ferroptosis regulation.

### DEGs and enrichment analysis of C17orf75 in LIHC

**Figure 4:**
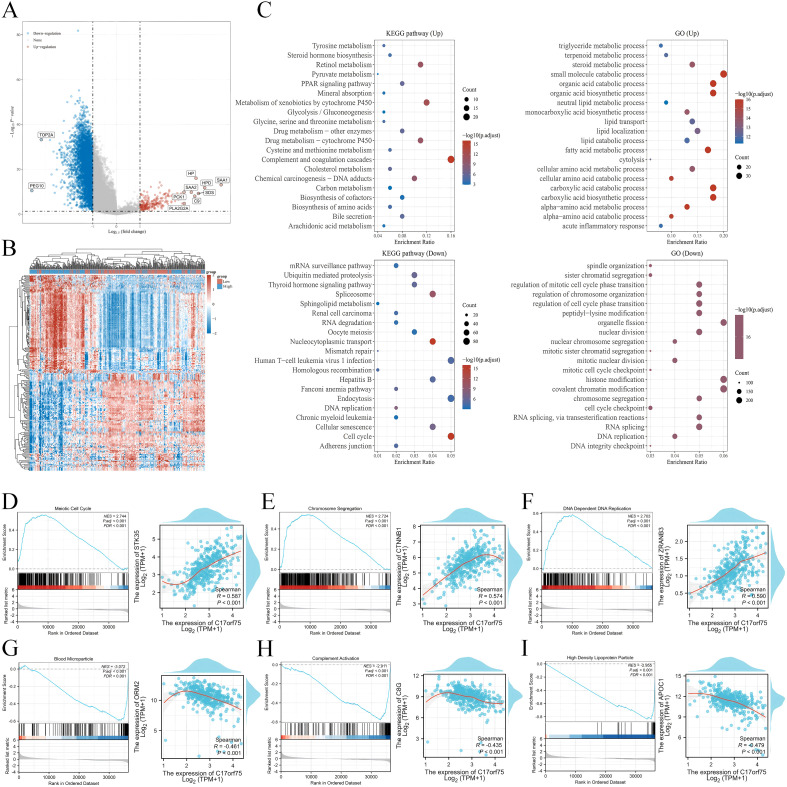
Analysis and enrichment analysis of C17orf75 differentially expressed genes (DEGs) in LIHC. **(A)** Volcano plot of LIHC DEGs. **(B)** Heatmap of DEGs in LIHC. **(C)** GO and KEGG enrichment analysis of C17orf75 DEGs in LIHC. **(D–I)** GSEA of C17orf75 in LIHC.

A total of 4,371 C17orf75-associated DEGs were identified in LIHC, including 196 upregulated and 4,175 downregulated genes (**Figures 4A, B**).

Upregulated genes were enriched in complement and coagulation cascades, whereas downregulated genes were enriched in cell cycle, endocytosis, and human T-cell leukemia virus 1 infection. GO analysis revealed that upregulated genes were enriched in small molecule catabolic process, organic substance catabolic process, and organic acid biosynthetic process, while downregulated genes were enriched in histone modification and covalent chromatin modification (**Figure 4C**).

GSEA further demonstrated that positively enriched pathways included cell cycle NES (Normalized enrichment scores) = 2.744, adjusted *p*-value [*p*.adj] < 0.001), chromosome segregation (NES = 2.724, *p*.adj < 0.001), and DNA-dependent DNA replication (NES = 2.703, *p*.adj < 0.001) (**Figures 4D–F**); whereas negatively enriched pathways included blood microparticle (NES = − 3.072, *P*.adj < 0.001), complement activation (NES = − 2.911, *P*.adj < 0.001), and high density lipoprotein particle (NES = − 2.955, *P*.adj < 0.001) (**Figures 4G–I**).

### Prognostic value of C17orf75 coexpressed genes and PPI network in LIHC

**Figure 5:**
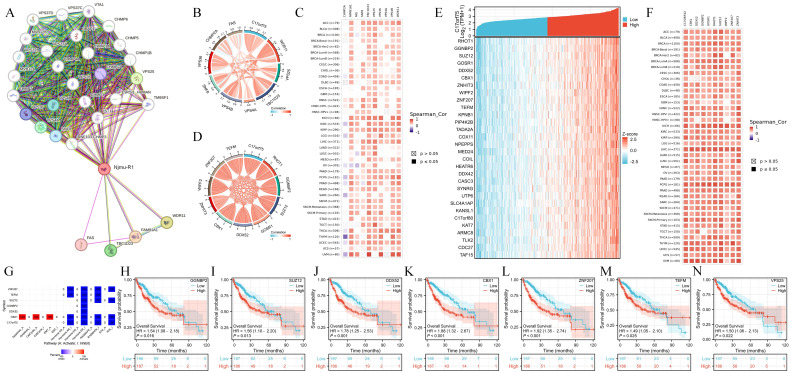
Coexpressed genes, prognostic value, and protein–protein interaction (PPI) network analysis of the protein encoded by C17orf75 (Njmu-R1). **(A)** PPI network analysis of the Njmu-R1 protein. **(B)** Correlation analysis of the top 10 PPI networks with C17orf75 in LIHC. **(C)** Heatmap of the top 10 associated genes in pan-cancer. **(D)** Correlation analysis of the top 10 coexpressed genes with C17orf75 in LIHC. **(E)** Heatmap of the top 30 genes with C17orf75 in LIHC. **(F)** Correlation heatmap of the top 10 coexpressed genes with C17orf75 across pan-cancer. **(G)** Enrichment analysis of C17orf75 and its related genes. **(H–N)** Prognostic value of C17orf75 coexpressed genes in LIHC.

We identified 30 proteins interacting with the protein encoded by C17orf75 (Njmu-R1) (**Figure 5A**). A correlation analysis was performed on the top 10 proteins (**Figure 5B**), and the expression correlations of these genes were examined across various cancers (**Figure 5C**).

We further analyzed coexpressed genes of C17orf75 in LIHC and identified the top 30 positively correlated genes (**Figures 5D, E**). Most of the top 10 coexpressed genes showed significant positive correlations with C17orf75 (**Figure 5F**).

Functional analysis using GSCALite indicated that these genes may regulate cell cycle, EMT, and hormone signaling (**Figure 5G**). Several coexpressed genes were significantly associated with poor prognosis in LIHC, including GGNBP2, SUZ12, DDX52, CBX1, ZNF207, TEFM, and VPS25 (Vacuolar protein sorting 25) (**Figures 5H–N**).

### Correlation between C17orf75 and TIME in pan-cancer

**Figure 6:**
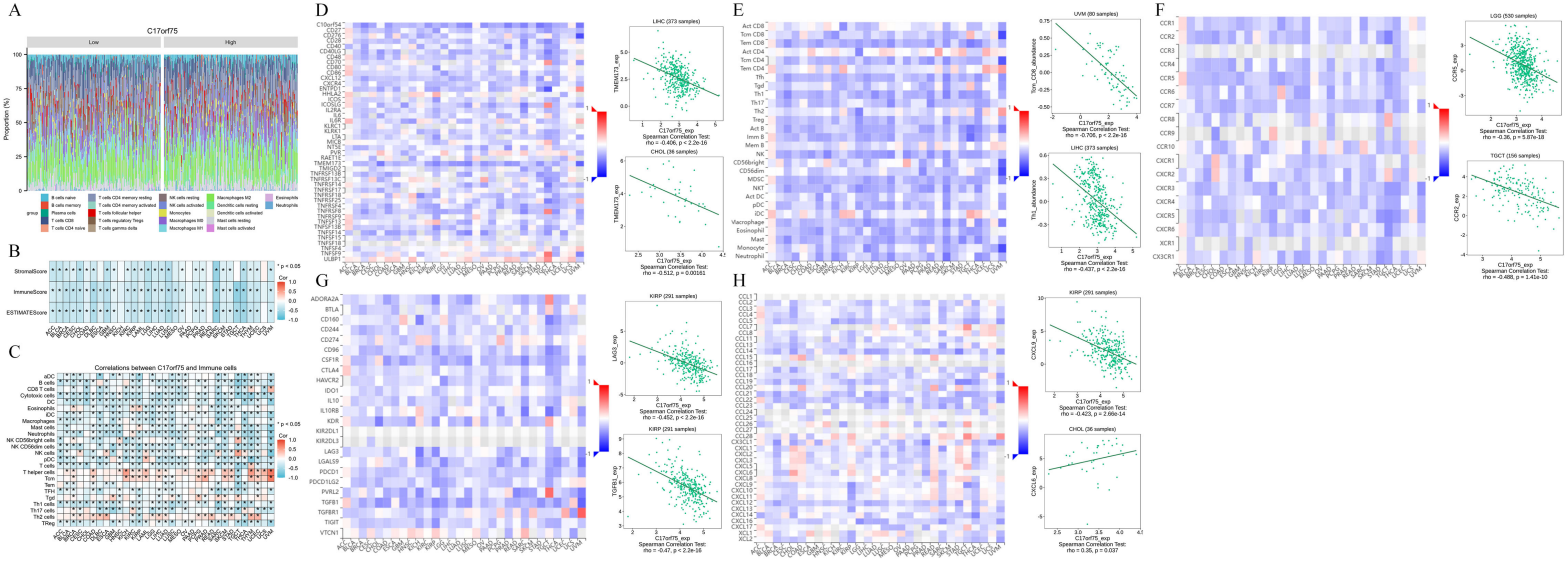
Immune infiltration analysis of C17orf75 expression in pan-cancer. **(A)** Distribution ratio of immune cells between high- and low-expression groups of C17orf75 in LIHC. **(B)** Correlation heatmap of C17orf75 expression with stromal, immune, and ESTIMATE scores. **(C)** Heatmap of the correlation of C17orf75 expression with immune cells via the ‘ssgsea’ algorithm. **(D–H)** Heatmaps of C17orf75 expression associations in the TISIDB database with **(D)** immune stimulators, **(E)** immune cells, **(F)** immune inhibitors, **(G)** chemokines, and **(H)** receptors.

In LIHC, T cells and macrophages were the dominant immune cell types (**Figure 6A**). Notably, high C17orf75 expression was generally associated with lower immune-related scores (**Figure 6B**). With the ‘ssgsea’ algorithm, we found that C17orf75 negatively correlated with the greatest number of immune cells throughout pan-cancer analyses (**Figure 6C**).

Further analysis using the TISIDB database revealed correlations between C17orf75 and immune-related factors, including immune stimulators, suppressors, chemokines, and receptors. C17orf75 negatively correlated with the immune stimulator TMEM173 in LIHC and CHOL (Cholangiocarcinoma) (**Figure 6D**), with Tcm_CD8 cells in UVM and Th1 cells in LIHC (**Figure 6E**), with the immune suppressors LAG3 and TGFB1 in KIRP (**Figure 6F**), and with the chemokine CXCL9 (Chemokine (C-X-C motif) ligand 9) in KIRP (Kidney renal papillary cell carcinoma). Conversely, it positively correlated with the chemokine CXCL6 in CHOL (**Figure 6G**) and showed a negative relationship with the receptors CCR5 (Chemokine receptor 5) in LGG and CCR2 in TGCT (**Figure 6H**).

### Single-cell expression of C17orf75 in LIHC

C17orf75 was predominantly expressed in proliferating T cells (Tprolif) in several datasets, while higher expression was also observed in hepatic progenitor cells, malignant cells, and NK cells (**Supplementary Figures 5A–H**).

### Drug sensitivity and molecular docking analysis of C17orf75

**Figure 7:**
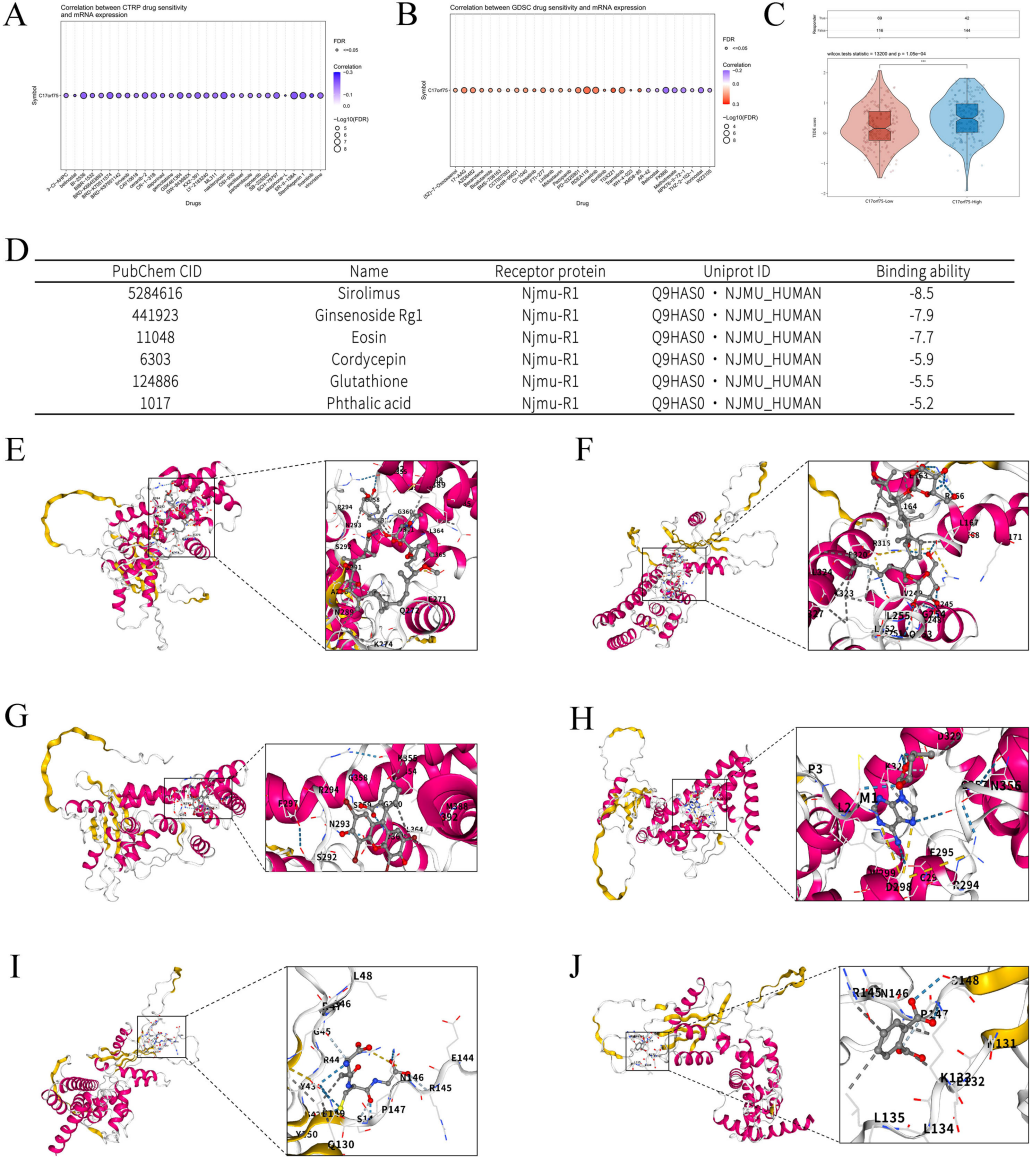
Chemotherapy drug sensitivity analysis of C17orf75 and molecular docking of Njmu-R1 protein. Analysis of C17orf75 expression and chemotherapy sensitivity based on CTRP **(A)** and GDSC **(B, C)** TIDE scores between high- and low-expression groups of C17orf75 in LIHC. **(D)** Overview of molecular docking of the Njmu-R1 protein. Molecular docking of sirolimus **(E)**, ginsenoside Rg1 **(F)**, eosin **(G)**, cordycepin **(H)**, glutathione **(I)**, and phthalic acid **(J)** with the Njmu-R1 protein.

Drug sensitivity analysis using CTRP and GDSC databases indicated that C17orf75 was negatively correlated with sensitivity to SR-II-138A, nakiterpiosin, BI-2536, BRD-K66453893, and StemRegenin 1, while positively correlated with RDEA119 (Refametinib), selumetinib, and trametinib (**Figures 7A, B**).

The low C17orf75 expression had significantly lower TIDE scores (*p* < 0.001), suggesting a better response to immunotherapy (**Figure 7C**).

Molecular docking analysis demonstrated that Njmu-R1 protein exhibited strong binding affinity with several compounds, including sirolimus, ginsenoside Rg1, eosin, cordycepin, glutathione, and phthalic acid (**Figures 7D–J**).

### Knockdown of C17orf75 attenuates the proliferation, migration, and invasion ability of hepatocellular carcinoma cells by arresting the cell cycle and inhibiting EMT

**Figure 8:**
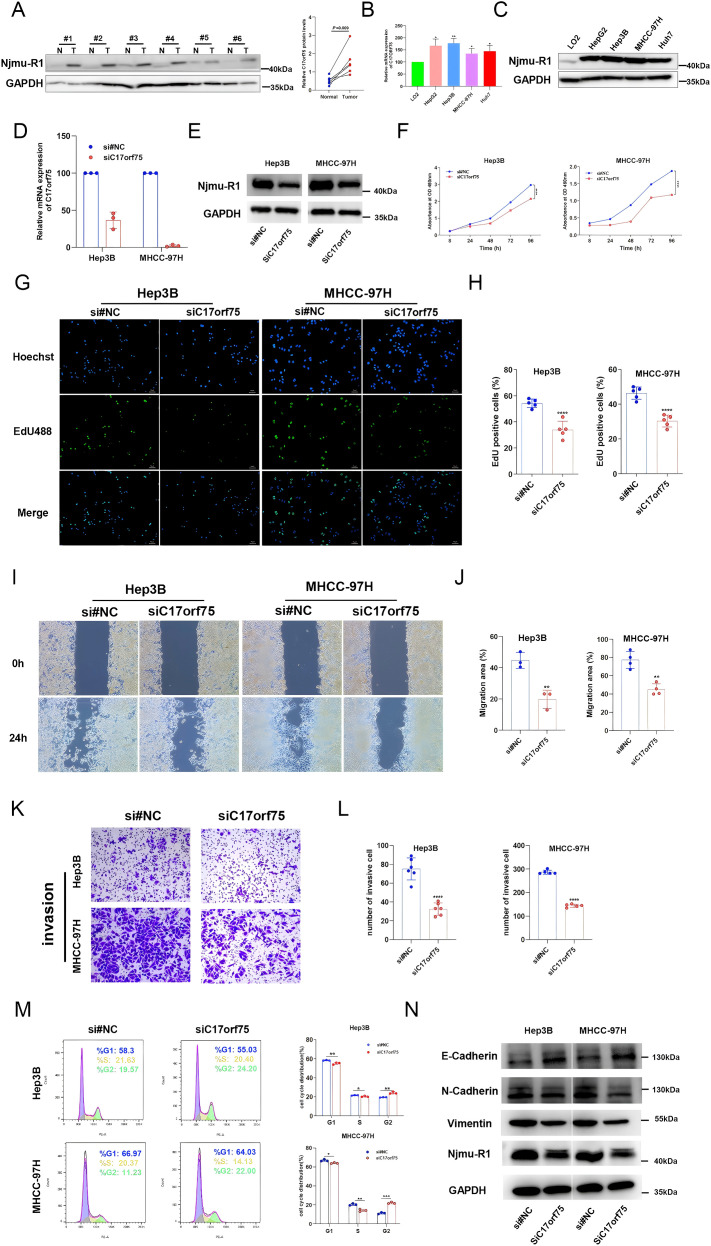
C17orf75 enhances proliferation, migration, and invasion of LIHC cells *in vitro*. **(A)** WB analysis of Njmu-R1 protein expression in tumor tissues. **(B, C)** Western blot analysis of Njmu-R1 protein and qRT-PCR analysis of C17orf75 mRNA expression in hepatocellular carcinoma cells. **(D, E)** Verification of C17orf75 knockdown efficiency. **(F–H)** C17orf75 knockdown inhibited proliferation. **(F–H)** EdU assays. Scale bar = 10 μm. **(I–L)** Knockdown of C17orf75 inhibited migration and invasion. **(M)** Knockdown of C17orf75 inhibited the cell cycle. **(N)** Knockdown of C17orf75 inhibited expression of EMT-associated proteins. (^*^p < 0.05; ^**^p < 0.01; ^***^p < 0.001; and ^****^p < 0.0001).

WB analysis revealed that Njmu-R1 protein levels were upregulated in LIHC tissues (**Figure 8A**). We performed IHC staining on 20 paraffin-embedded LIHC tissue samples with complete clinical and pathological data. Njmu-R1 protein expression was higher in tumor tissues, and Njmu-R1 expression was found to be positively correlated with tumor stage, with higher expression levels observed in advanced-stage tumors (**Supplementary Figure 6A**). C17orf75 (Njmu-R1) was highly expressed in HepG2, Hep3B, MHCC-97H, and Huh7 cells, whereas its expression was significantly lower in LO2 cells (**Figures 8B, C**).

We downregulated C17orf75 expression in MHCC-97H and Hep3B cells. As shown, the siRNAs efficiently downregulated C17orf75 mRNA levels and Njmu-R1 protein levels (**Figures 8D, E**).

C17orf75 knockdown significantly suppressed the growth of both Hep3B and MHCC-97H cells over 96 h in CCK-8 assays (**Figure 8F**). Similar results were observed in the EdU assay. Quantitative analysis showed that the proliferation rate of these cells was decreased by approximately 30%–40% after C17orf75 knockdown (**Figures 8G, H**).

Wound-healing assays revealed that C17orf75 silencing significantly attenuated wound healing capacity in Hep3B and MHCC-97H cells (**Figures 8I, J**). For the invasion assay, C17orf75 knockdown notably reduced the number of invasive Hep3B and MHCC-97H cells. The migratory and invasive abilities of LIHC cells were reduced by approximately 40%–60% after C17orf75 knockdown (**Figures 8K, L**).

The flow cytometry results showed that knockdown of C17orf75 induced G2/M phase cell cycle arrest in both Hep3B cells (19.57% to 24.20%) and MHCC-97H cells (11.23% to 22.00%) (**Figure 8M**). No significant effect on apoptosis was observed upon C17orf75 knockdown (**Supplementary Figure 6B**). To determine whether Njmu-R1 modulates EMT in LIHC cells, we examined classic EMT markers by Western blotting following C17orf75 knockdown. Compared with siNC controls, E−cadherin was clearly upregulated in both Hep3B and MHCC−97H cells upon C17orf75 knockdown, whereas N−cadherin and vimentin were markedly decreased (**Figure 8N**).

Collectively, these data demonstrate that C17orf75 (Njmu-R1) plays a critical role in promoting the proliferation, migration, and invasion of LIHC cells. C17orf75 may simultaneously regulate cell proliferation and metastasis-related phenotypes, key processes that drive tumor progression and metastasis.

## Discussion

In this study, we systematically investigated the role of C17orf75 across pan-cancer using integrated bioinformatics analyses and experimental validation. We explored the prognostic and diagnostic value of C17orf75 and examined the mutation landscape of C17orf75. We performed enrichment analysis of DEGs in LIHC, constructed a PPI network based on the protein encoded by C17orf75 (Njmu-R1), and evaluated its prognostic significance. We analyzed the association between C17orf75 and immune infiltration in a pan-cancer, evaluated its association with response to immunotherapy and targeted therapy, and explored its potential involvement in ferroptosis and m6A methylation. This study has revealed the role of C17 in pan-cancer, particularly in LIHC.

As previously reported, the biological and diagnostic significance of C17orf75 has not been fully characterized. According to the GeneCards database, C17orf75 is highly expressed in the pancreas, placenta, bone, and skin, which is consistent with our findings. C17orf75 demonstrated strong diagnostic performance in multiple cancers, including CHOL, COAD, LIHC, LUSC, and STAD. Prognostic heatmap analyses indicated that high C17orf75 expression was associated with poor OS, DSS, and PFI in LIHC.

The ClinVar database indicates that C17orf75 harbors SNVs at specific loci, potentially leading to amino acid substitutions, although their clinical significance remains unclear (36). C17orf75 alterations occur in approximately 3% of pan-cancer cases, with amplification being the most common type. These alterations may influence gene expression and contribute to tumor development (37).

To further explore the molecular mechanisms of C17orf75, we analyzed DEGs and co-expressed genes. Upregulated genes were enriched in pathways related to metabolism, DNA replication, and cell cycle. Tumorigenesis requires dysregulation of glucose and amino acid uptake, alterations in the regulation driven by metabolic genes, and effective adaptation to adverse conditions in the tumor microenvironment (TME) through metabolic reprogramming, including hypoxia, severe oxidative stress, acidic pH, and nutrient deprivation (38). Carcinogenic DNA replication stress can lead to heritable DNA damage, which is transmitted to daughter cells via mitosis, thereby paving the way for incomplete DNA replication within large replication complexes in the human genome (39). We hypothesize that C17orf75 may be involved in the pathogenesis of LIHC by interacting with molecules in these pathways and regulating the associated signaling complexes.

PPI network data support this view: with the exception of CHMP2A (Chromatin-modifying protein 2a),, key genes such as FAM91A1, FAS (Fas cell surface death receptor), SNF8 (SNF8 Subunit Of ESCRT-II), TBC1D23, VPS25, VPS36, VPS4A, VPS4B, and WDR11 all show a positive correlation and potential interactions with the protein encoded by C17orf75 (Njmu-R1). Among these core genes, CHMP2A is a targetable antitumor immunosuppressive factor; following knockout of this gene via CRISPR/Cas9 technology, head and neck squamous cell carcinoma (HNSC) becomes more sensitive to NK cell-mediated killing (40). Tolerance to COAD immunotherapy is associated with impaired FAS-dependent apoptosis (41). Biallelic mutations in SNF8 lead to a range of neurodevelopmental and neurodegenerative disorders (42). TBC1D23 acts by activating the β1 integrin/FAK/ERK downstream pathway, promoting lung cancer cell proliferation (43). VPS25 promotes immune evasion in HNSC by increasing the number of PVRs (Poliovirus receptors) in cancer cells, thereby activating the immunosuppressive PVR-TIGIT axis (44). In COAD, depletion of VPS4A and VPS4B proteins induces cell death while releasing immunomodulatory factors that regulate inflammation and antitumor responses (45). These interactions further support the potential involvement of C17orf75 in vesicle trafficking and tumor-related signaling pathways.

Cancer cells undergo morphological and functional changes, thereby acquiring the ability to invade surrounding tissues and distant organs (46). We found that C17orf75 and its core genes are jointly enriched in apoptosis, cell cycle, EMT, and hormone signaling. C17orf75 may play a critical role in regulating tumor progression through multiple biological processes, prompting further exploration of its underlying mechanisms.

Intracellular trafficking plays a crucial role in tumor progression by regulating the transport, localization, and turnover of key signaling molecules. Dysregulation of vesicle trafficking has been shown to affect cell cycle progression, receptor signaling, and EMT, thereby contributing to cancer cell proliferation and metastasis. In the present study, the observed effects of C17orf75 on G2/M phase arrest and EMT-related markers suggest that it may exert its oncogenic functions, at least in part, through modulation of intracellular trafficking processes. One possible mechanism is that C17orf75 influences the trafficking or recycling of growth factor receptors or adhesion molecules, thereby altering downstream signaling pathways.

In addition, intracellular trafficking is closely associated with the Golgi apparatus function, which is essential for protein processing and secretion. Although the involvement of Golgi-related mechanisms was not directly examined in this study, it is plausible that C17orf75 may participate in Golgi-dependent transport processes. Further studies are required to clarify these mechanisms. In particular, given its potential role in intracellular vesicle trafficking and protein complex regulation, the protein encoded by C17orf75 (Njmu-R1) may influence the spatial organization or activation of signaling molecules, thereby exerting broad regulatory effects on tumor cell behavior. However, whether these regulatory mechanisms are conserved across different cancer types or are specific to LIHC remains to be determined.

The TME impedes T-cell activation and anticancer activity by locally depleting glucose, glutamine, other amino acids, and specific lipids. The accumulation of specific immunosuppressive metabolites also impairs the T-cell immune response. Choi et al. demonstrated that SH003 and DTX enhance antitumor immunity by increasing the infiltration of cytotoxic T cells and NK cells (47–49). We found a significant positive correlation between C17orf75 and helper T cells and Tcm cells in LIHC. We therefore hypothesize that elevated levels of C17orf75 may help cancer cells evade immune attack by suppressing the generation or abundance of monocytes and inhibiting the activity of macrophages, DCs, and NK cells, thereby ultimately promoting tumorigenesis and metastasis.

Currently, ICI therapy for LIHC shows great promise, with several clinical trials—including those involving nivolumab and pembrolizumab—already underway (50). Our study suggests that lower levels of C17orf75 expression are associated with improved ICI efficacy in LIHC. Trametinib, as a reversible inhibitor of mitogen-activated protein kinase 1 (MEK1), regulates the MAPK signaling pathway by targeting the MEK protein, thereby inhibiting cell proliferation and producing an anti-LIHC therapeutic effect (51). Our study found that upregulation of C17orf75 is positively correlated with enhanced response to drugs such as RDEA119, cerulimetinib, and trametinib, suggesting that C17orf75 may serve as a potential target for precision chemotherapy in tumors. Furthermore, although the Njmu-R1 protein interacts with various molecules, its specific mechanisms and clinical significance require further validation through further research.

Notably, rather than affecting a single cellular process, our findings indicate that C17orf75 may coordinate multiple oncogenic programs, including cell cycle progression and EMT, thereby promoting LIHC progression. This integrative effect distinguishes it from many genes identified through bioinformatics analyses that are often limited to descriptive associations.

In summary, our findings highlight C17orf75’s potential role in tumor progression through coordinated regulation of multiple oncogenic processes and suggest that it may become a promising biomarker. Nevertheless, further studies are required to elucidate the precise molecular mechanisms, particularly those involving the Njmu-R1 protein, in tumor development and immune regulation. 

## Data Availability

The bioinformatics data presented in the study are publicly available online, and the raw experimental data can be provided upon reasonable request.

## Supplementary material

Supplementary Figure 1 Expression of C17orf75 in normal human organs and tissues. **(A)** Overview of mRNA expression of C17orf75 and protein expression of Njmu-R1. **(B-D)** Expression patterns of C17orf75 mRNA in different tissues from consensus, GTEx, and HPA databases. **(E)** Distribution of Njmu-R1 protein expression. **(F)** Global expression overview of C17orf75 mRNA at the single-cell level. **(G)** Global expression overview of C17orf75 mRNA in LIHC cell lines. **(H)** Subcellular localization of Njmu-R1 protein. **(I)** Protein structure of Njmu-R1. **(J)** Subcellular localization of Njmu-R1 protein visualized by immunofluorescence.

Supplementary Figure 2 C17orf75 mutation analysis. **(A)** Mutation status of C17orf75. **(B)** Alteration frequency and mutation types of C17orf75. **(C)** Visualization of C17orf75 mutation sites. **(D)** Heatmap of C17orf75 CNV percentage. **(E)** Distribution of homozygous CNVs in C17orf75. **(F)** Distribution of heterozygous CNVs in C17orf75. **(G)** Percentage heatmap of SNVs in C17orf75. **(H)** Summary of variant distribution clusters by variant classification, type, and SNV category in C17orf75. **(I)** Summary of transition (Ti) and transversion (Tv) classifications for SNVs in C17orf75. **(J)** Lollipop plot of C17orf75 mutation distribution in LIHC. **(K)** Summary of variant distribution clusters by variant classification, type, and SNV category in C17orf75 across LIHC. **(L)** Oncoplot of somatic mutation landscape in LIHC samples. (**P* < 0.05).

Supplementary Figure 3 Diagnostic value of C17orf75 in pan-cancer. **(A)** ROC curve of C17orf75. **(B)** Time-dependent ROC curve of C17orf75.

Supplementary Figure 4 Correlation analysis of C17orf75 with m6A methylation and ferroptosis in LIHC. **(A, B)** Heatmaps showing correlations between C17orf75 and genes associated with m6A methylation and ferroptosis.

Supplementary Figure 5 Expression of C17orf75 in the immune microenvironment of LIHC.

Supplementary Figure 6 Validation of Njmu-R1 protein expression in clinical LIHC samples by IHC and impact of C17orf75 on apoptosis by Flow Cytometry. **(A)** Representative images of Njmu-R1 protein expression across different tumor stages and adjacent normal tissues, Scale bar = 50 μm. **(B)** The effect of C17orf75 knockdown on apoptosis of LIHC cells.
