# Supplementary figures and images for: C17orf75 (Njmu-R1) promotes hepatocellular carcinoma progression: a pan-cancer analysis and experimental validation
Source: Front Immunol. 2026 Jul 9;17:1828437. doi: 10.3389/fimmu.2026.1828437 (PMC13391555; doi:10.3389/fimmu.2026.1828437)

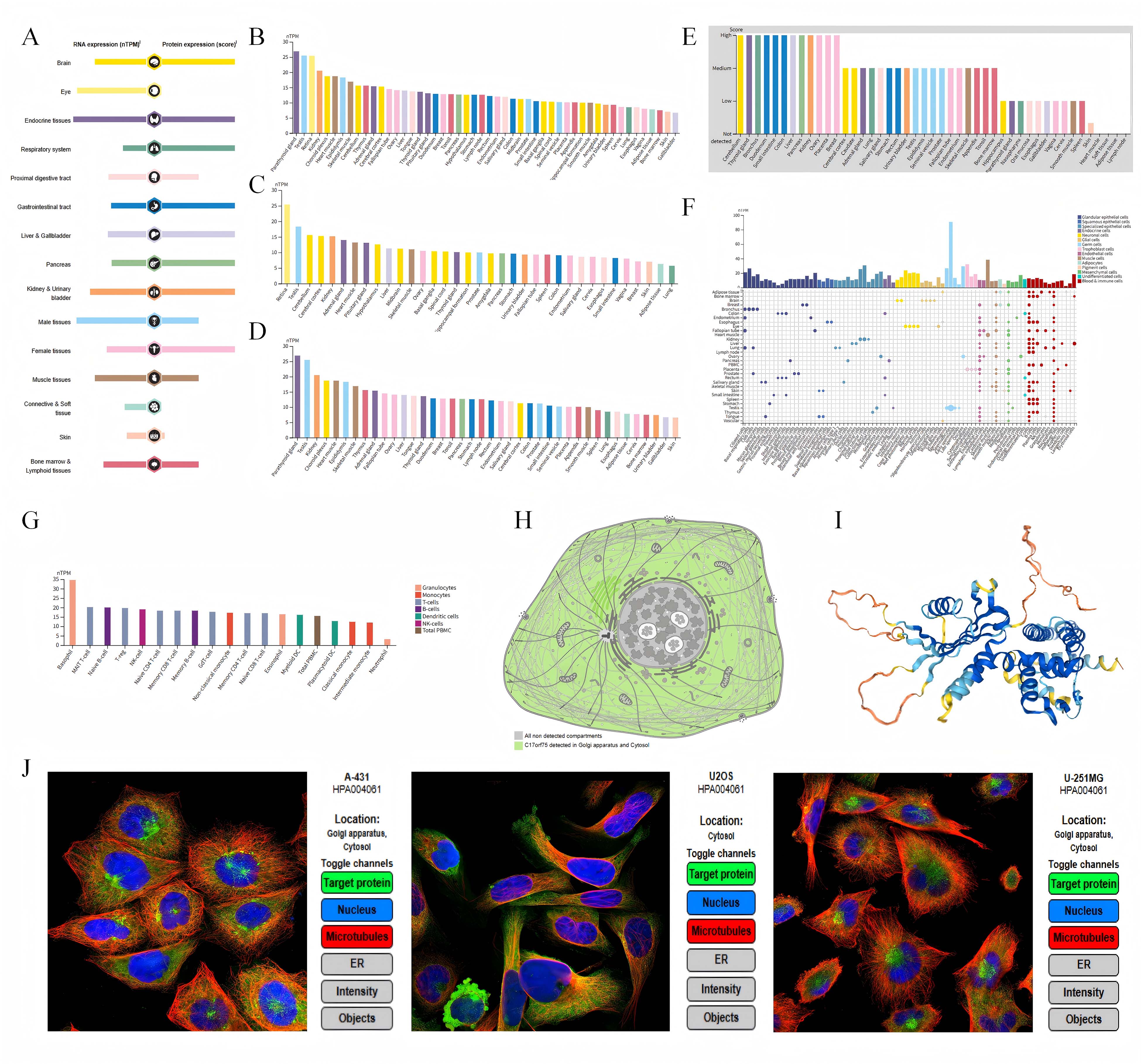

Supplement: Supplementary Figure 1 — Expression of C17orf75 in normal human organs and tissues. (A) Overview of mRNA expression of C17orf75 and protein expression of Njmu-R1. (B-D) Expression patterns of C17orf75 mRNA in different tissues from consensus, GTEx, and HPA databases. (E) Distribution of Njmu-R1 protein expression. (F) Global expression overview of C17orf75 mRNA at the single-cell level. (G) Global expression overview of C17orf75 mRNA in LIHC cell lines. (H) Subcellular localization of Njmu-R1 protein. (I) Protein structure of Njmu-R1. (J) Subcellular localization of Njmu-R1 protein visualized by immunofluorescence. [file Image1.jpg]

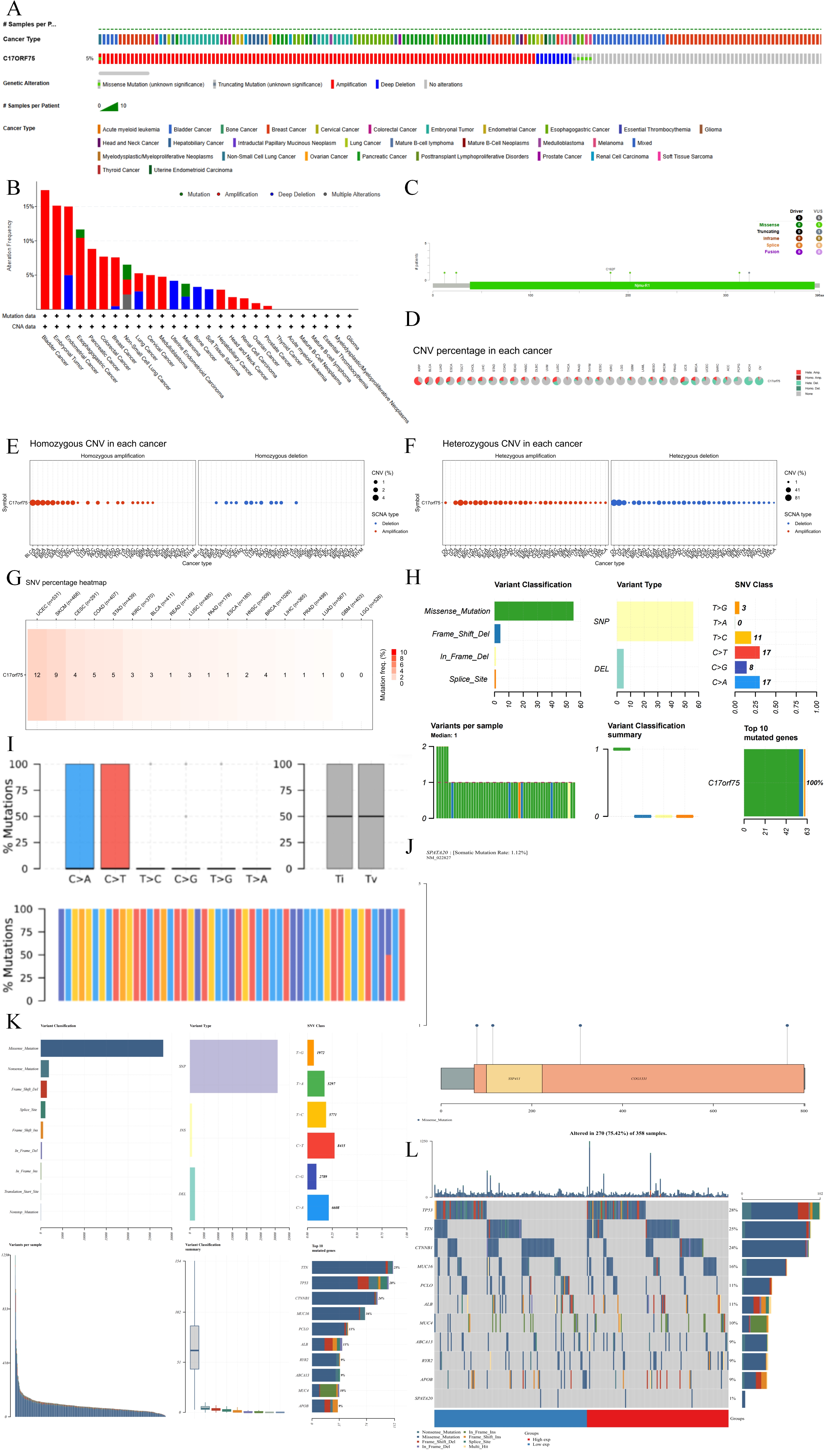

Supplement: Supplementary Figure 2 — C17orf75 mutation analysis. (A) Mutation status of C17orf75. (B) Alteration frequency and mutation types of C17orf75. (C) Visualization of C17orf75 mutation sites. (D) Heatmap of C17orf75 CNV percentage. (E) Distribution of homozygous CNVs in C17orf75. (F) Distribution of heterozygous CNVs in C17orf75. (G) Percentage heatmap of SNVs in C17orf75. (H) Summary of variant distribution clusters by variant classification, type, and SNV category in C17orf75. (I) Summary of transition (Ti) and transversion (Tv) classifications for SNVs in C17orf75. (J) Lollipop plot of C17orf75 mutation distribution in LIHC. (K) Summary of variant distribution clusters by variant classification, type, and SNV category in C17orf75 across LIHC. (L) Oncoplot of somatic mutation landscape in LIHC samples. (*P < 0.05). [file Image2.jpeg]

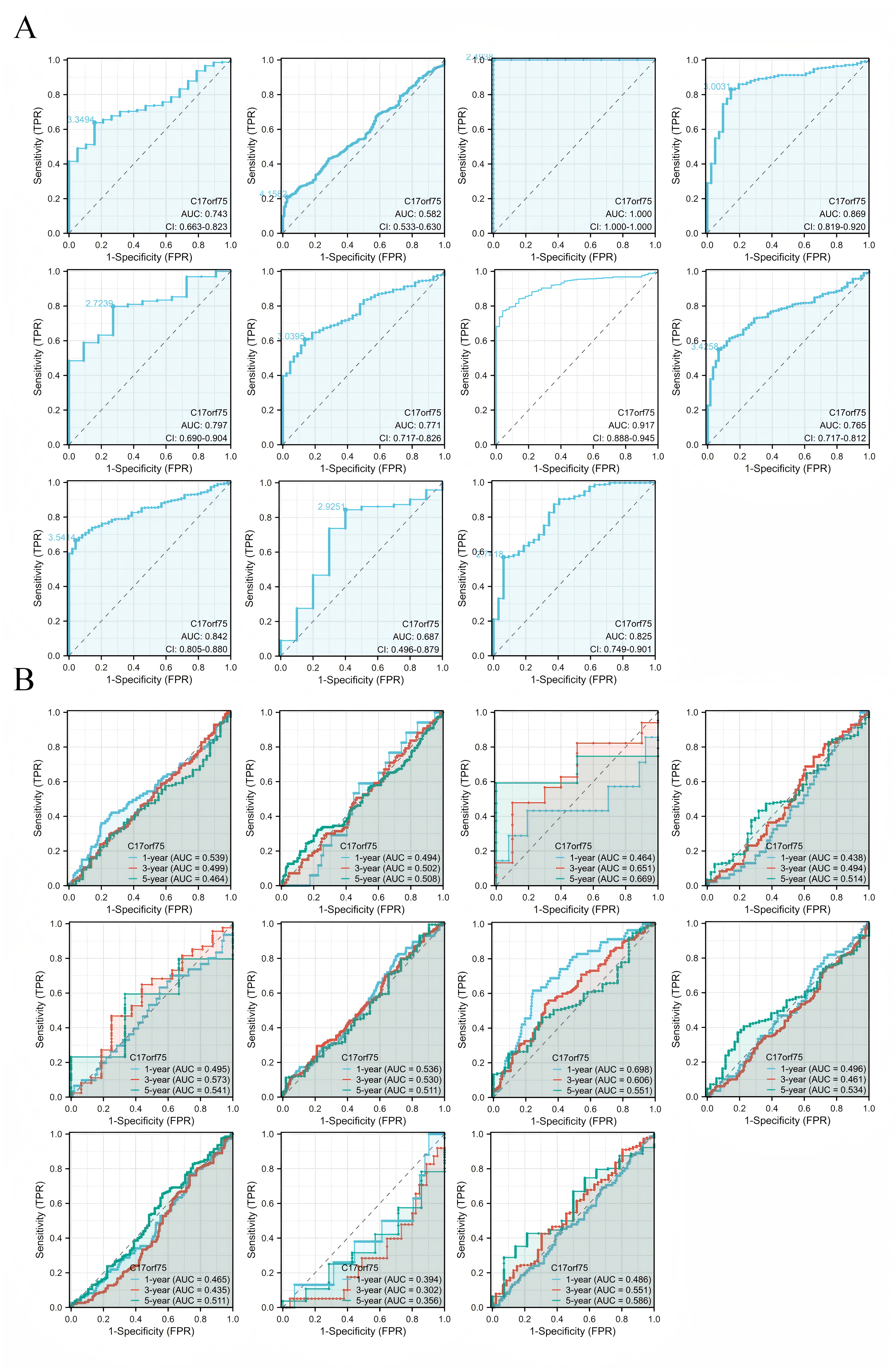

Supplement: Supplementary Figure 3 — Diagnostic value of C17orf75 in pan-cancer. (A) ROC curve of C17orf75. (B) Time-dependent ROC curve of C17orf75. [file Image3.jpeg]

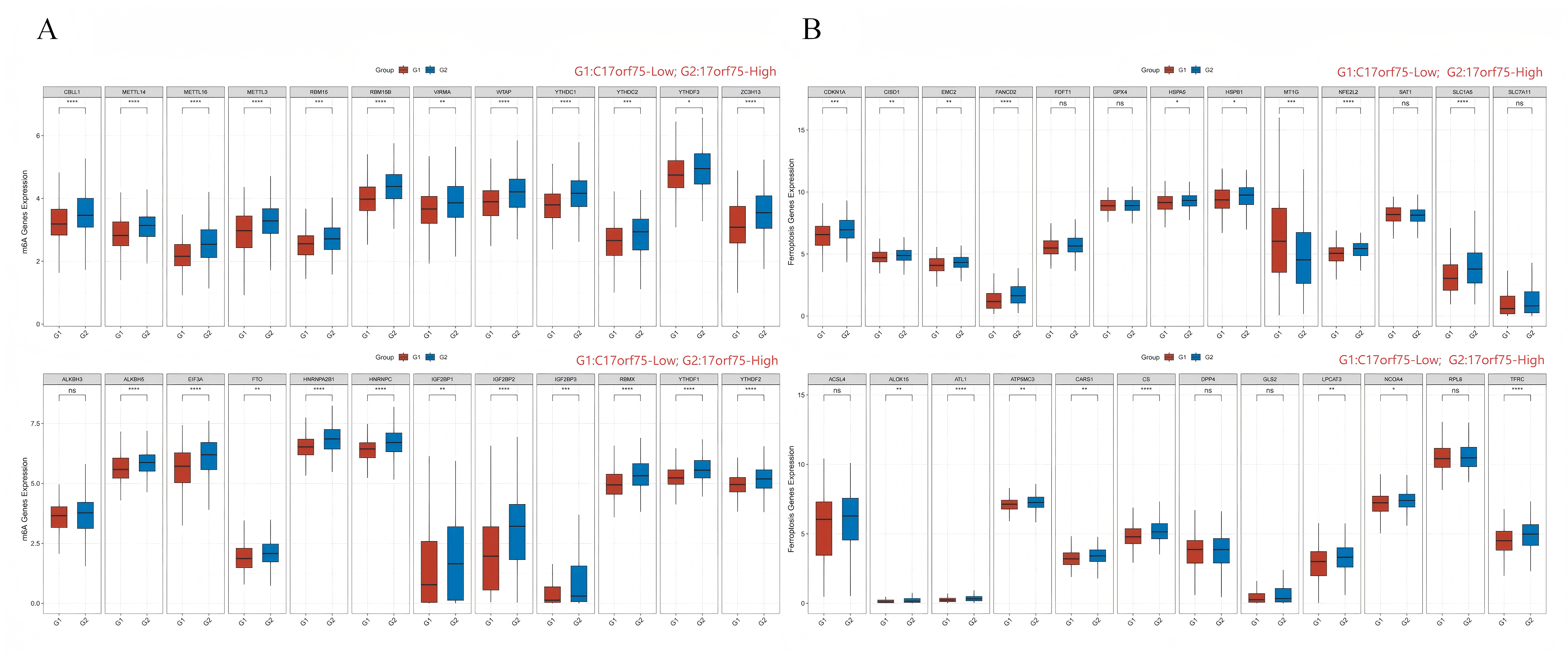

Supplement: Supplementary Figure 4 — Correlation analysis of C17orf75 with m6A methylation and ferroptosis in LIHC. (A, B) Heatmaps showing correlations between C17orf75 and genes associated with m6A methylation and ferroptosis. [file Image4.jpeg]

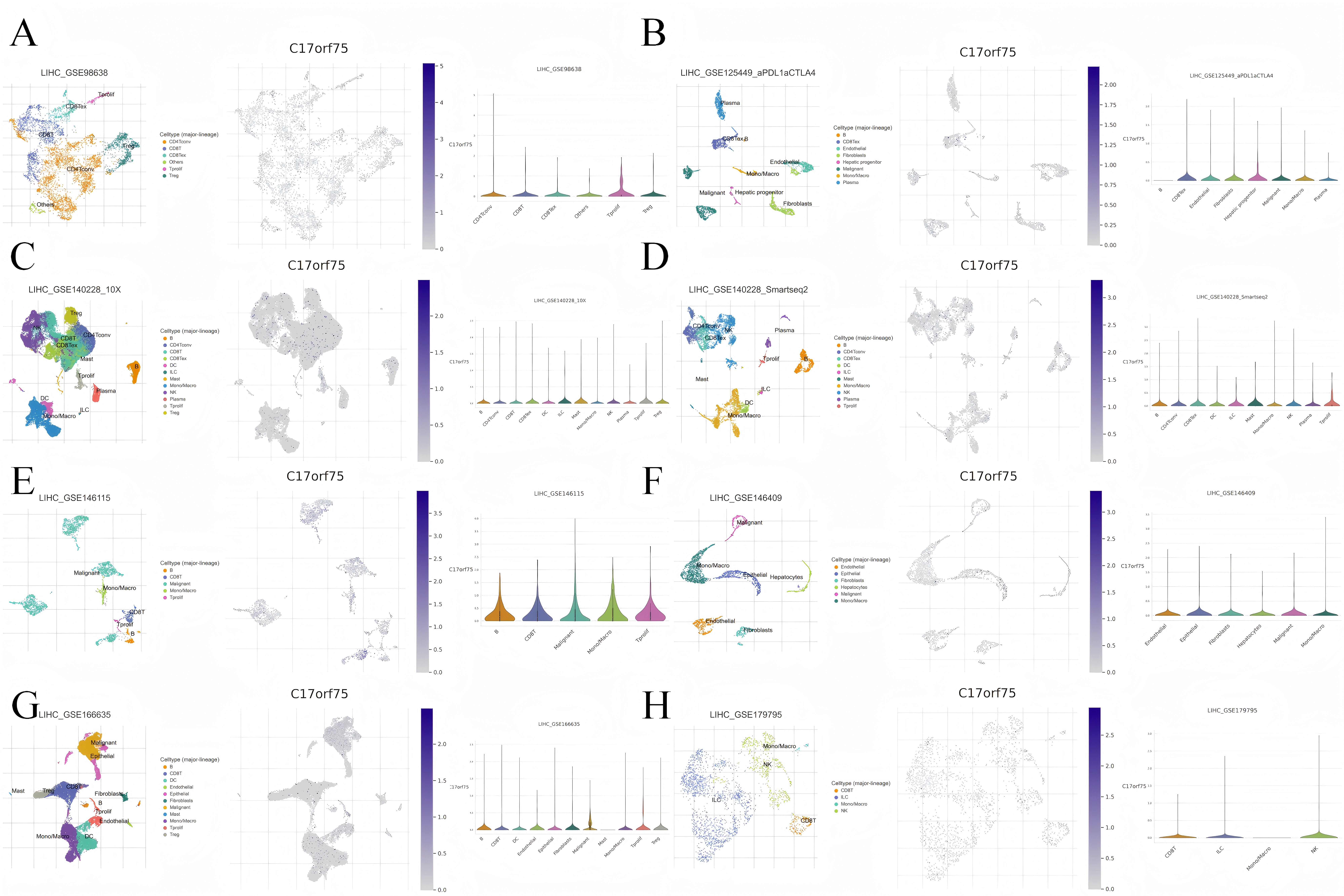

Supplement: Supplementary Figure 5 — Expression of C17orf75 in the immune microenvironment of LIHC. [file Image5.jpeg]

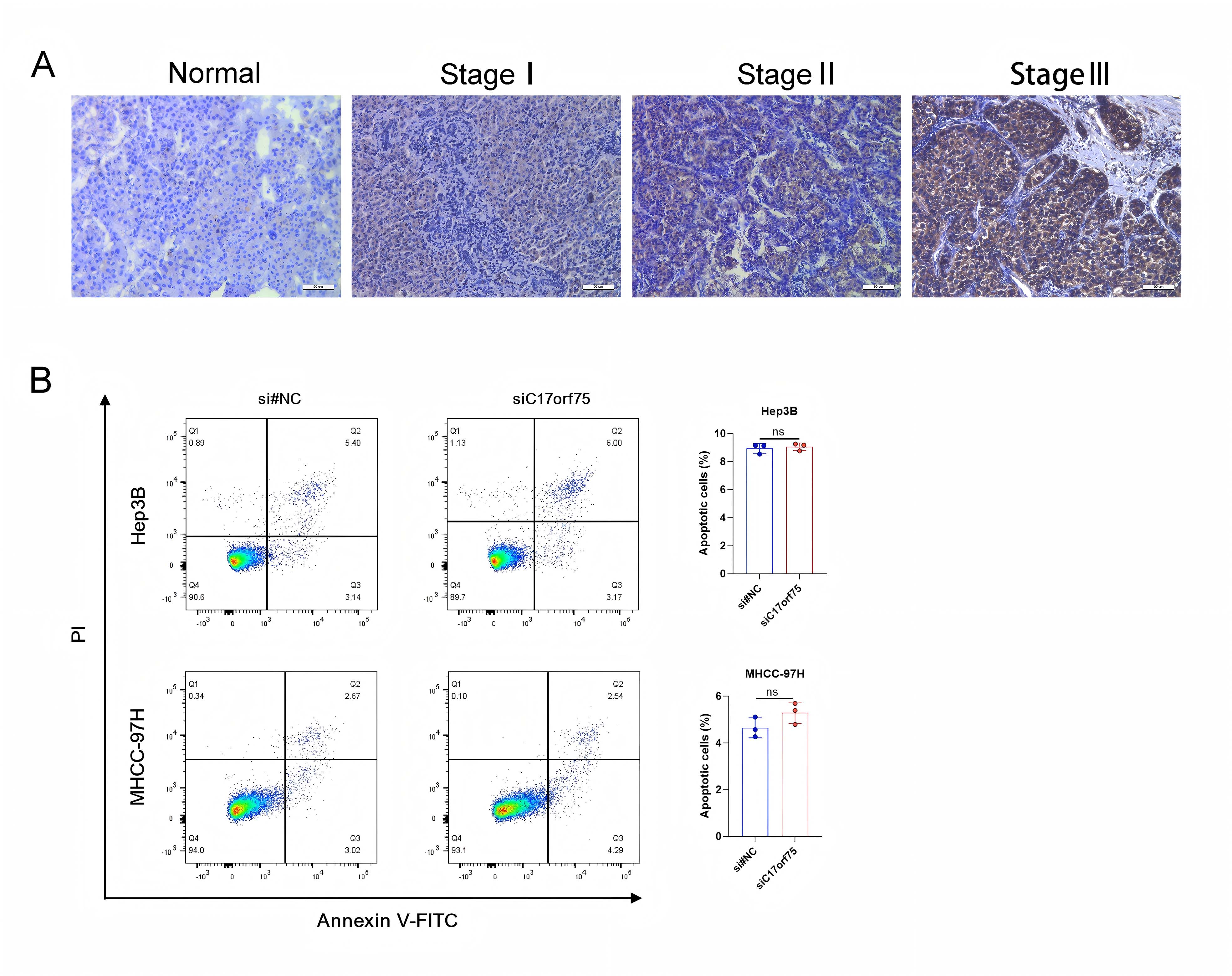

Supplement: Supplementary Figure 6 — Validation of Njmu-R1 protein expression in clinical LIHC samples by IHC and impact of C17orf75 on apoptosis by Flow Cytometry. (A) Representative images of Njmu-R1 protein expression across different tumor stages and adjacent normal tissues, Scale bar = 50 μm. (B) The effect of C17orf75 knockdown on apoptosis of LIHC cells. [file Image6.jpg]
