# Supplementary material for: C17orf75 (Njmu-R1) promotes hepatocellular carcinoma progression: a pan-cancer analysis and experimental validation
Source: Front Immunol. 2026 Jul 9;17:1828437. doi: 10.3389/fimmu.2026.1828437 (PMC13391555; doi:10.3389/fimmu.2026.1828437)
Supplement: Supplementary file 7 [file SupplementaryFile1.docx]

**Methods**

**Data Collection and Processing**

RNA expression data of C17orf75 across normal human tissues were obtained from the Human Protein Atlas (HPA) database, including the HPA, GTEx, and consensus datasets.RNA sequencing data were also collected to evaluate C17orf75 expression in immune cells and single-cell datasets. In addition, subcellular localization data derived from immunofluorescence experiments were collected. Protein localization and expression patterns of Njmu-R1 were further obtained from IHC data, along with corresponding annotation information [12].

RNA sequencing data and clinical information for multiple cancer types were downloaded from The Cancer Genome Atlas (TCGA) database[13]. Tumor samples and matched adjacent normal tissues were included, while samples lacking complete clinical information were excluded. The “ggplot2” R package was used to visualize differences in C17orf75 expression across tumors, normal tissues, pan-cancer cell lines, LIHC cell lines, and paired tumor-normal samples.

Gene expression datasets GSE19804 and GSE37182 were obtained from the Gene Expression Omnibus (GEO) database for validation analysis. Spatial transcriptomics data were retrieved from the SpatialTME database, including four LIHC samples (HCC_JH-HCC_P8, HCC_Pmid35673582-HCC1, HCC_Pmid35673582-HCC2, and HCC_Pmid35673582-HCC3). Spatial distribution analysis was performed to evaluate differences in C17orf75 expression between tumor and normal tissues[14].

**Patients and tissue samples**

20 pairs of LIHC specimens, including paired tumor and adjacent non-tumorous tissues as well as formalin-fixed paraffin-embedded samples, were collected for validation experiments.

**Mutation landscape of C17orf75**

The cBioPortal database was used to analyze the mutation landscape of C17orf75 across pan-cancer, including alteration frequency, mutation types, and mutation sites. For LIHC, RNA sequencing data, mutation annotation format (MAF) files, and corresponding clinical information were downloaded from TCGA[15]. Somatic mutation data were visualized using the “maftools” R package[16]. Copy number variation (CNV) analysis of C17orf75 was performed using the GSCA database[17].

**Diagnostic and Prognostic Analysis**

We evaluated the intercorrelation between C17orf75 expression and clinicopathological characteristics. Receiver operating characteristic (ROC) curves were constructed using the “pROC” package to assess diagnostic performance.

Univariate Cox regression analysis was performed using the “survival” package to evaluate the association between C17orf75 expression and overall survival (OS), disease-specific survival (DSS), and progression-free interval (PFI)[18]. LIHC patients were divided into high- and low-expression groups based on the median expression level of C17orf75. Kaplan–Meier survival analysis was conducted using the “survival” package. A prognostic nomogram was constructed using the “rms” package, and calibration curves were generated to evaluate prediction accuracy.

**Clinicopathological Correlation Analysis**

we performed logistic regression analysis to evaluate the association between C17orf75 expression and clinicopathological features in LIHC. The Wilcoxon rank-sum test or Kruskal–Wallis test was used to compare differences among groups. Variables included tumor stage, histological grade, AFP level, vascular invasion, and other clinical characteristics.

**Ferroptosis and m6A Analysis**

The correlation between C17orf75 expression and ferroptosis- and m6A-related genes was analyzed. Ferroptosis-related genes were obtained from published literature, and m6A regulators were selected based on previous studies[19-22]. .

**DEGs and Enrichment Analysis**

LIHC samples were divided into C17orf75 high- and low-expression classes. Differential expression investigation was accomplished with the ‘limma’ package. The threshold was set at |log2FC| > 1.5 and adjusted p-value < 0.05. Functional enrichment analysis, including GO, KEGG, and GSEA, was performed using the “clusterProfiler” package[23].

**PPI Network and Co-expression Analysis**

The protein–protein interaction (PPI) network of the protein encoded by C17orf75 (Njmu-R1) was constructed using the STRING database[24]. The top correlated genes were identified based on expression correlation and interaction scores. TIMER2 was used to analyze gene expression patterns across cancers[25]. The prognostic significance of hub genes and pathway enrichment were further analyzed using GSCALite[26].

**Tumor Immune Microenvironment Analysis**

We analyzed RNA-seq data using the CIBERSORT algorithm to estimate the relative proportions of immune cell types between high- and low-C17orf75 expression groups in LIHC. The ‘ESTIMATE’ algorithm was applied to calculate immune scores, stromal scores, and ESTIMATE scores [27].

The “corrplot” package was used to visualize the correlations among these scores across cancer types. In addition, the TISIDB database was used to assess the associations between C17orf75 expression and immune infiltration across multiple cancer types [28]. These immune-related factors included immune checkpoints, immune cells, immunostimulators, immunoinhibitors, chemokines, and their receptors.

**Single-Cell Expression Analysis**

Single-cell RNA-seq data (in .h5 format) and LIHC annotation files were obtained from the TISCH database[29].

**Drug Sensitivity and Molecular Docking Analysis**

We selected the ‘pRRophetic’ package for chemotherapy sensitivity prediction, with half-maximal inhibitory concentration (IC50) scores were estimated using ridge regression models [30].

The Tumor Immune Dysfunction and Exclusion (TIDE) algorithm was applied to predict potential responses to immune checkpoint inhibitor (ICI) therapy [31].

Potential small-molecule compounds targeting C17orf75 were identified using the Coremine database [32]. Structural information for the Njmu-R1 protein encoded by C17orf75 was obtained from the Protein Data Bank (PDB) database [33]. Ligand structures were downloaded from the PubChem database, and molecular docking simulations were performed using CB-Dock2 to evaluate binding interactions between Njmu-R1 and candidate small-molecule compounds [34].

**Cell culture and transfection**

Human hepatocellular carcinoma cell lines (HepG2, Hep3B, MHCC-97H, and Huh7) and the normal hepatocyte cell line LO2 were obtained from ATCC or the China Center for Type Culture Collection. Cells were cultured in DMEM supplemented with 10% FBS and 1% penicillin–streptomycin at 37 °C with 5% CO₂.

siRNAs targeting C17orf75 (siC17orf75) and a negative control (siNC) were synthesized by Sangon Biotech. MHCC-97H and Hep3B cells were transfected using Lipofectamine 3000 according to the manufacturer’s instructions. After 48 h, cells were collected for subsequent analyses.

The related siRNA sequences are as follows:

si#NC forward: (5′-UUCUCCGAACGUGUCACGUdTdT-3′),

si#NC reverse: (5′-ACGUGACACGUUCGGAGAAdTdT-3′);

siC17orf75 forward: (5′-GGAACUAGAGAGCAGCGA AdTdT-3′),

siC17orf75 reverse: (5′-UUCGCUGCUCUCUAGUUC CdTdT-3′);

**Quantitative Real-Time PCR and western blot analysis**

Total RNAs were extracted using an RNAiso Plus Kit (Takara, Japan) and reverse-transcribed using a PrimeScript™ RT Master Mix Kit (Takara, Japan). Quantitative real-time PCR analysis (qRT-PCR) was conducted using CFX96^TM^ Real-time PCR system (Bio-Rad, CA, USA) and a TB Green kit (Takara, Japan) to determine C17orf75 mRNA expression levels. Gene expression data were analyzed via the 2⁻^ΔΔCt^ method, GAPDH acted as the internal control for normalization. The primer sequences used in this study were listed below.

C17orf75:(forward:5′-CTGAGCAGCTGGTTTGAGGA3′; reverse: 5′-TCCTTGAAGACTGGCCACAC-3′)

GAPDH:(forward:5′-GTCTCCTCTGACTTCAACAGCG-3′; reverse: 5′-ACCACCCTGTTGCTGTAGCCAA-3′)

Proteins from tissues and cells were extracted in radioimmunoprecipitation assay (RIPA) lysis buffer (P0013, Beyotime, China) with protease and phosphatase inhibitors (CWBIO, China). The identical amounts of proteins were electrophoresed by 10% SDS-PAGE, transferred onto 0.2 μm PVDF membranes, and incubated with primary antibody against the Njmu-R1 protein (1:1000, Proteintech, China, Cat#26326-1-AP), E-cadherin (1:20000, Proteintech, China, Cat#20874-1-AP), N-cadherin (Proteintech, China, Cat#222018-1-AP), Vimentin (Proteintech, China, Cat#80232-1-AP) and GAPDH (1:5000, Proteintech, China, Cat#10494-1-AP) at 4 °C overnight, followed by incubation with appropriate HRP-conjugated secondary antibody (1:2000, Cell Signaling Technology, USA ) at room temperature for 1 h. Signals were detected by Immobilon ECL substrate (Bio-Rad, USA).

## **Immunohistochemistry**

Paraffin-embedded LIHC tissue sections (n = 20) from different tumor stages were subjected to immunohistochemical staining. After deparaffinization, rehydration, and antigen retrieval, sections were incubated with a primary antibody against the Njmu-R1 protein (1:200, Proteintech, China, Cat#26326-1-AP), followed by incubation with an HRP-conjugated secondary antibody. Staining was visualized using DAB and counterstained with hematoxylin. Njmu-R1 protein expression was evaluated based on staining intensity and the proportion of positive cells.

**Cell counting kit (CCK)-8 and EdU assays**

Cell proliferation was evaluated using CCK-8 and EdU assays according to the manufacturer’s instructions. For the CCK-8 assay, cells were seeded in 96-well plates, and absorbance at 450 nm was measured after incubation with CCK-8 reagent. For the EdU assay, cells were incubated with EdU, fixed, and stained, followed by imaging under a fluorescence microscope. EdU-positive cells were quantified using ImageJ.

**Wound healing assays**

Cells were seeded into culture inserts and allowed to reach confluence. After removing the inserts, cells were cultured in complete medium, and wound closure was observed at 24 h under a microscope.

**Matrigel invasion assays**

Cell invasion was assessed using Matrigel-coated Transwell chambers. Cells were seeded in the upper chamber with serum-free medium, while complete medium was added to the lower chamber. After incubation, invaded cells were fixed, stained, and counted under a microscope.

**Cell Cycle and Apoptosis Analysis by Flow Cytometry**

Cell cycle and apoptosis were analyzed by flow cytometry. For cell cycle analysis, cells were fixed and stained with propidium iodide. For apoptosis analysis, cells were stained with Annexin V-FITC. Data were analyzed using FlowJo software.

**Statistical analysis**

Statistical analyses were conducted using R (v4.5.1) and GraphPad Prism (v8.0). Data distribution was evaluated for normality with the Shapiro–Wilk test. All experiments were carried out in triplicate, and results are presented as mean ± standard deviation (SD). For comparisons between two groups, Student’s t-test was applied to normally distributed data, whereas the Wilcoxon signed-rank test was used when normality was not met. When analyzing three or more groups, one-way ANOVA followed by Dunnett’s post hoc test was used for parametric data; nonparametric data were assessed using the Kruskal–Wallis test with Bonferroni adjustment. Associations between variables were examined using Spearman’s rank correlation coefficient. A P value < 0.05 was considered indicative of statistical significance. Significance thresholds are reported as follows: *P* < 0.05, *P* < 0.01, *P* < 0.001, and *P* < 0.0001; “ns” denotes not significant (*P* ≥ 0.05).
